# Thrombolytic efficacy and enzymatic activity of rt-PA-loaded echogenic liposomes

**DOI:** 10.1007/s11239-015-1204-8

**Published:** 2015-04-02

**Authors:** Kenneth B. Bader, Guillaume Bouchoux, Tao Peng, Melvin E. Klegerman, David D. McPherson, Christy K. Holland

**Affiliations:** 1 Division of Cardiovascular Health and Disease, Department of Internal Medicine, College of Medicine, Cardiovascular Center 3933, University of Cincinnati, Cincinnati, OH USA; 2 Biomedical Engineering Program, University of Cincinnati, Cincinnati, OH USA; 3 Division of Cardiovascular Medicine, Department of Internal Medicine, University of Texas Health Science Center-Houston, Houston, TX USA

**Keywords:** Acute ischemic stroke, Ultrasound, Ultrasound contrast agents, Acoustic cavitation, Echogenic lipsomes

## Abstract

Echogenic liposomes (ELIP), that can encapsulate both recombinant tissue-type plasminogen activator (rt-PA) and microbubbles, are under development to improve the treatment of thrombo-occlusive disease. However, the enzymatic activity, thrombolytic efficacy, and stable cavitation activity generated by this agent has yet to be evaluated and compared to another established ultrasound-enhanced thrombolytic scheme. A spectrophotometric method was used to compare the enzymatic activity of the rt-PA incorporated into ELIP (t-ELIP) to that of rt-PA. An in vitro flow model was employed to measure the thrombolytic efficacy and dose of ultraharmonic emissions from stable cavitation for 120-kHz ultrasound exposure of three treatment schemes: rt-PA, rt-PA and the perfluorocarbon-filled microbubble Definity^®^, and t-ELIP. The enzymatic activity of rt-PA incorporated into t-ELIP was 28 % that of rt-PA. Thrombolytic efficacy of t-ELIP or rt-PA and Definity^®^ was equivalent when the dose of t-ELIP was adjusted to produce comparable enzymatic activity. Sustained bubble activity was nucleated from Definity but not from t-ELIP exposed to 120-kHz ultrasound. These results emphasize the advantages of encapsulating a thrombolytic and the importance of incorporating an insoluble gas required to promote sustained, stable cavitation activity.

## Introduction

Stroke is currently the fourth leading cause of death in the United States of America [1], and a leading cause of death worldwide [2]. The only FDA-approved thrombolytic drug, recombinant tissue-type plasminogen activator (rt-PA), is administered only in 1.5 % of cases [1] due to potential bleeding complications and strict exclusion criteria [3]. Thus, targeted delivery of this potent thrombolytic is an attractive concept, with implications for treatment of thrombo-occlusive disease.

Echogenic liposomes (ELIP) containing gas microbubbles have been developed as a vector for therapeutic drugs [4]. Thrombolytic-loaded liposomes, termed t-ELIP, target fibrin [5], and can be acoustically activated [6, 7] for localized drug delivery. Encapsulation of rt-PA may reduce systemic toxicity compared to direct intravenous injection of this potent thrombolytic. Furthermore, ultrasound insonation of the gas microbubbles within t-ELIP can potentially release the rt-PA locally and incite stable cavitation [8]. Stable cavitation activity is known to be correlated with ultrasound enhancement of rt-PA thrombolysis [9, 10]. Thus, t-ELIP has the potential to target the clot, deliver rt-PA locally, and enhance thrombolytic efficacy.

Thrombolytic-loaded t-ELIP have been shown to enhance thrombolysis compared to rt-PA in vitro in a static fluid system [6, 11]. A limitation of the in vitro models employed by Shaw et al. and Tiukinhoy-Laing et al. was the absence of flow, which may influence both thrombolytic efficacy [12] and cavitation activity [13]. Equivalent thrombolytic efficacy of t-ELIP and rt-PA has been demonstrated in an in vivo rabbit model [14]. The type and amount of cavitation activity nucleated by 120-kHz insonation of t-ELIP, however, has not previously been investigated.

The objective of this study was therefore to explore the relationship between thrombolytic efficacy, cavitation emissions, and the enzymatic activity of rt-PA-loaded ELIP. Here, an in vitro flow model [10, 15] was used to study thrombolytic efficacy and stable cavitation emissions in real-time during t-ELIP sonothrombolysis. The maximum lytic rate, time to 50 % clot width reduction, and percent reduction in clot width over 30 min were used as metrics of thrombolytic efficacy. In addition, the enzymatic activity of rt-PA was measured before and after its loading into ELIP.

## Materials and methods

### Preparation of human fresh frozen plasma and rt-PA

Human fresh-frozen plasma (hFFP) was procured from a blood bank (Hoxworth Blood Center, Cincinnati, OH). Thirty milliliter aliquots of the hFFP were thawed for each experiment and allowed to reach atmospheric gas equilibrium at 37 °C in an open container for 2 h. rt-PA was obtained from the manufacturer (Activase^®^, Genentech, San Fransisco, CA, USA) as lyophilized power. Each vial was mixed with sterile water to a concentration of 1 mg/mL as per manufacturer instructions, aliquoted into 1.0 mL centrifuge tubes, and stored at −80 °C. The enzymatic activity of rt-PA is stable over a period of 7 years using this protocol [16].

### Preparation of blood clots

Human whole blood clots were manufactured around silk sutures following a protocol developed by Shaw et al. [17]. Following local Institutional Review Board approval and written informed consent, venous human whole blood was drawn from a pool of 10 healthy volunteers. Aliquots of 500 μL were transferred to sterile glass tubes containing borosilicate glass micropipettes (1.12 mm inner diameter, World Precision Instruments, Inc., USA), pre-threaded with 7-0 silk sutures (Ethicon Industries, Cornelia, GA). The blood was allowed to clot around the silk suture at 37 °C for 3 h. Following clot formation, the tubes were stored at 5 °C for a minimum of 3 days to allow for maximal clot retraction [6], lytic resistance, and stability [18]. Before each measurement, the micropipette was removed to produce a cylindrical clot adherent to the suture. The initial clot size (550 ± 43 μm) was smaller than that of the middle cerebral artery (2.4–4.6 mm) [19], the site of occlusion for the majority of ischemic strokes [20]. However, the clot size is comparable to the perforating branches of the middle cerebral artery (80–840 μm) [21], which are highly vulnerable to occlusion.

### Preparation of ultrasound contrast agents (UCAs)

Vials of Definity^®^ (perflutren lipid microspheres; Lantheus Medical Imaging, N. Billerica, MA, UCA), containing octofluoropropane encapsulated microbubble by a lipid monolayer shell, were activated according to the manufacturer’s instructions. The vial was stored at 5 °C until needed. The vial was allowed to warm to room temperature (20–24 °C) for one hour prior to activation by shaking for 45 s using a Vial-Mix^®^ (Lantheus Medical Imaging). The agent was diluted to a final concentration of 2 μL/mL (1 × 10^4^ particles/mL). This number density is consistent with the manufacturer’s recommended dose (Lantheus Medical Imaging, Billerica, MA, USA) for left ventricular opacification.

### Preparation of rt-PA loaded echogenic liposomes

rt-PA-loaded ELIP (t-ELIP) were prepared at the University of Texas Health Science Center, Houston, as described by Huang et al. [22] and shipped to the University of Cincinnati on dry ice. Each vial containing 2 mg of lyophilized lipids and 80 μg of rt-PA was reconstituted with 0.2 μL of 0.2 μm filtered deionized water (NANOPure, Barnstead International, Dubuque, IA, USA), and diluted in hFFP within 5 min of reconstitution to achieve rt-PA concentrations of 0.32, 1.58, and 3.15 μg/mL (lipid concentrations of 8.0, 39.5, and 78.7 μg/mL, respectively).

### rt-PA activity measurement

The enzymatic activity of rt-PA and t-ELIP were measured using a spectrophotometric method [7, 23]. Thawed rt-PA or reconstituted t-ELIP were diluted into a solution of 0.5 % BSA and 1 % Triton-X (Sigma-Aldrich, St. Louis, MO, USA) to achieve concentrations of rt-PA between 0.3 and 3 μg/mL in disposable cuvettes used for spectrophotometric measurement. The amount of Triton-X employed in the solution exceeded the critical micelle concentration (0.015 %) (Triton-X Product Information Sheet, Sigma-Aldrich, St. Louis, MO, USA) to ensure rupture of the lipid shell surrounding t-ELIP and release of the associated rt-PA. The solution was aspirated to remove echogenic microbubbles [24], and diluted into a pre-warmed solution (37 °C) of 0.5 % phosphate buffer solution and a chromogenic substrate (S-2288, Chromogenix, DiaPharma Group, Inc., Westchester, OH, USA). The chromogenic substrate is hydrolyzed by rt-PA and allows spectrophotometrical measurement of the change of absorbance at 405 nm over time, which is proportional to the enzymatic activity of rt-PA. A spectrophotometer (UV-1700, Shimadzu, Japan) with temperature controller (TCC-240A, Shimadzu, Japan) was used to record the absorbance of the solution over the course of 5 min at 37 °C. The rt-PA activity was reported in terms of the change in absorbance over time (ΔAbs/min).

### In vitro flow phantom

**Fig. 1:**
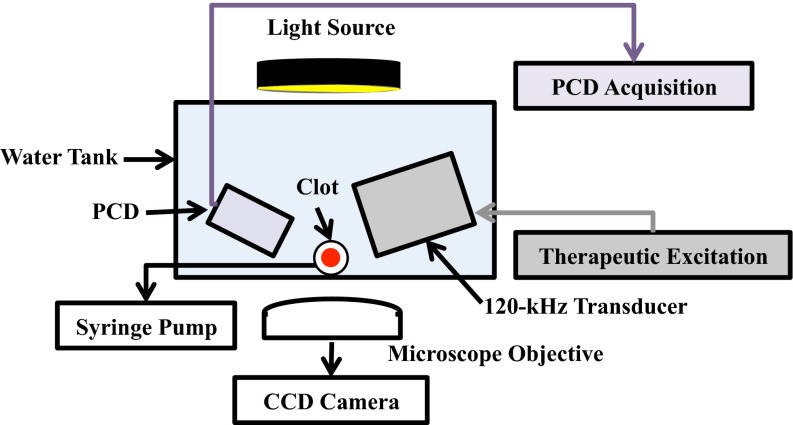
Side view of experimental set for observations of ultrasound-enhanced thrombolysis. Flow is out of the page

The in vitro flow model depicted in Fig. 1 was based on Cheng et al. [25] and Gruber et al. [15], and was used to quantify thrombolytic efficacy and stable cavitation activity. An acrylic tank (16 × 33 × 9 cm) was filled with approximately 3 L of degassed (20 ± 5 % dissolved oxygen), reverse-osmosis water heated to 37.3 ± 0.3 °C. The water was continuously filtered (0.2 μm), and the gas content and temperature were maintained throughout the experiment with a custom-built recirculation system. The walls of the tank were lined with a 1 cm thick acoustic absorber (Aptflex F48, Precision Acoustics, Dorchester, Dorset, UK).

The flow channel consisted of low-density polyethylene tubing (inner diameter 1.6 mm, outer diameter 3.2 mm, part 1 J-109-10, Freelin Wade Co., McMinnville, OR, USA) to direct the plasma and therapeutics from a reservoir to a glass micropipette (2.15 mm inner diameter, 0.3 mm wall thickness 5-000-2200, Drummond Scientific Co., Broomall, PA, USA). A clot was mounted along the central axis of the micropipette by snuggly fitting the suture at the ends of the micropipette with latex tubing. The micropipette was positioned over a microscope slide (Fisherbrand, 12-550C, Fisher Scientific, Pittsburg, PA, USA) on the bottom of the tank to allow imaging of the clot with an inverted microscope (IX71, Olympus Corporation, Center Valley, PA, USA). The focal area of the objective (UPlanFLN 10X, 10 mm working distance, Olympus Corporation, Center Valley, PA, USA) was approximately 1200 × 900 μm. Images were captured by a CCD camera (Retiga-2000R, Q Imaging, Surrey, BC, Canada) at a rate of 2.33 Hz. Flow was maintained at 0.65 mL/min with a programmable syringe pump (model 44, Harvard Apparatus Co. Inc., South Natick, MA, USA) in continuous withdrawal mode. This flow rate is in the range of physiologic flow rates measured in the occluded middle cerebral artery during ischemic stroke [26].

### Ultrasound exposure and cavitation detection

A custom-designed 120-kHz transducer (H160, Sonic Concepts, Inc. Woodburn, WA, USA) was used to insonate the clot and the perfusate within the micropipette. The unfocused transducer element (30 mm diameter aperture) was excited at its resonant frequency of 120 kHz with a function generator (33250A, Agilent Technologies, Inc., Santa Clara, CA, USA) and power amplifier (1040L, ENI, Rochester, NY, USA). A custom-built impedance matching network (Sonic Concepts, Inc. Woodburn, WA, USA) maximized power transfer to the transducer. The acoustic field was measured and the in situ acoustic pressure calibrated along the clot with a 0.5-mm hydrophone (TC 4038, Teledyne Reson Inc. Goleta, CA, USA) mounted on a computer-controlled three-axis positioner (NF-90, Velmex Inc., Bloomfield, NY, USA).

Ultraharmonic (UH) emissions, a key acoustic signature of stable cavitation [27], were monitored with a passive cavitation detector (PCD) aligned confocal with the clot. The PCD, a 19-mm-diameter circular single-element, long-focus 2.25-MHz transducer (595516C, Picker Roentgen GmbH, Espelkamp, Germany), has previously been utilized to detect cavitation generated by 120-kHz insonation [10, 27]. The signal received from the PCD was filtered by a 10-MHz low-pass filter (J73E, TTE Inc, Los Angeles, CA, USA) to remove noise from radiofrequency interference, and amplified with a wideband low-noise amplifier (CLC100, Cadeka Microcircuits LLC, Colorado, USA). The signal was digitized (10 ms duration, 31.25 MHz sampling frequency), and the power spectrum computed in MATLAB^®^ (The Mathworks, Natick, MA, USA). UH bands [13] of the power spectrum between 250 kHz and 1 MHz were summed over a 2-kHz bandwidth centered around each UH. The choice of these frequency bands for the characterization of stable cavitation activity was detailed in a previous study [15]. The UH dose for a given experiment is defined here as the integration of the UH power detected over the 30 min experiment duration.

An intermittent ultrasound exposure scheme, as described previously [13], was employed to maximize the UH dose over the 30 min treatment duration. A peak-to-peak pressure of 0.44 MPa and insonation period of 50 s were found to maximize UH emissions over the 30 min treatment duration [10]. The insonation period was followed by a 30 s quiescent period to allow a fresh influx of Definity^®^ or t-ELIP to fill the micropipette. Insonation and quiescent periods were continuously repeated during the 30 min treatment.

### Experimental procedure

A clot was mounted in the capillary tube, and submerged in the temperature-controlled fluid within the tank. The position of the tank was adjusted so that the clot could be visualized by the microscope objective. The focus of the PCD was aligned with the capillary tube. Clots were treated for 30 min. The experiments were distributed into 3 controls: (1) plasma alone; (2) plasma and optimized intermittent ultrasound exposure (0.44 MPa peak-to-peak continuous wave, 50 s on, 30 s off); and (3) plasma plus Definity^®^ (2 μL/mL) and optimized intermittent ultrasound exposure and 5 treatment types: (1) rt-PA alone; (2) t-ELIP alone; (3) rt-PA and the optimized intermittent ultrasound scheme; (4) rt-PA and Definity^®^ and the optimized intermittent ultrasound scheme; and (5) t-ELIP and the optimized intermittent ultrasound scheme. For each treatment, rt-PA concentrations of 0, 0.32, 1.58, and 3.15 μg/mL were investigated. These rt-PA concentrations are within the therapeutic concentration range in humans [28]. Images and acoustic emissions recorded by the PCD were acquired at a rate of 2.33 Hz (0.43 s inter-frame time), and stored for analysis offline. A total of 12 experiments (using clots from four different donors) were performed for a given treatment type and concentration of rt-PA (180 total experiments).

### Clot diameter determination

Images of the clots were used to determine thrombolytic efficacy [25]. The clot edges were tracked using an edge-detection routine following Meunier et al. [29]. The clot diameter for a given frame, $\bar{d}$, was defined as the average distance between the detected edges for each row of the image (600 rows total, 900 μm total length), minus the diameter of the suture (95 ± 15 μm).

The fractional clot loss, *FCL*, was defined as the percent reduction of the clot diameter at the conclusion of the measurement compared to the initial clot diameter:

1 $$ FCL = 100\, \times \,\frac{{\bar{d}\left( {t_{0} } \right) - \bar{d}(t_{30} )}}{{\bar{d}(t_{0} )}} $$

where $\bar{d}(t)$ is the clot diameter at time $t$, the subscript ‘0’ indicates the initial time point, and the subscript ‘30’ indicates the 30 min time point. The time-dependent reduction in clot width, $\varLambda (t)$, was fit in the least squares sense to a sigmoidally-decreasing function [30]:

2 $$ \varLambda \left( t \right) = 100 - \frac{100}{{1 + e^{{ - \beta (t - t_{50} )}} }} $$

where $\beta$ and $t_{50}$ are fitting parameters. The time to a 50 % reduction in clot width occurs at $t = t_{50}$. The maximum slope (i.e. the lytic rate) of Eq. (1) occurs as $t$ approaches $t_{50}$. Using a Taylor expansion of Eq. (2) around $|t - t_{50} | \ll 1$, the maximum lytic rate, $LR_{MAX}$, can be determined as:

3 $$ LR_{MAX} = 25\beta . $$

### Statistical analysis

Statistical analysis was performed using the MATLAB Statistical Toolbox (The Mathworks, Natick, MA, USA). Statistical differences in means of the fractional clot loss, maximum lytic rate, and time to 50 % clot width reduction between treatment types at each concentration of rt-PA were determined by one-way unbalanced ANOVA with an *α* level of 0.05. Adjusted *p* values based on Tukey’s HSD test are reported to elucidate significant differences between the means of treatment cohorts. The correlation between the fractional clot loss at 30 min, the maximum lytic rate, and the time to 50 % clot width reduction and rt-PA concentration were calculated using the Spearman’s rank correlation coefficient in MATLAB^®^ (The Mathworks, Natick, MA, USA). A *p* < 0.05 was used as a requirement for significant correlation.

## Results

### Functional form for reduction in clot width

**Fig. 2:**
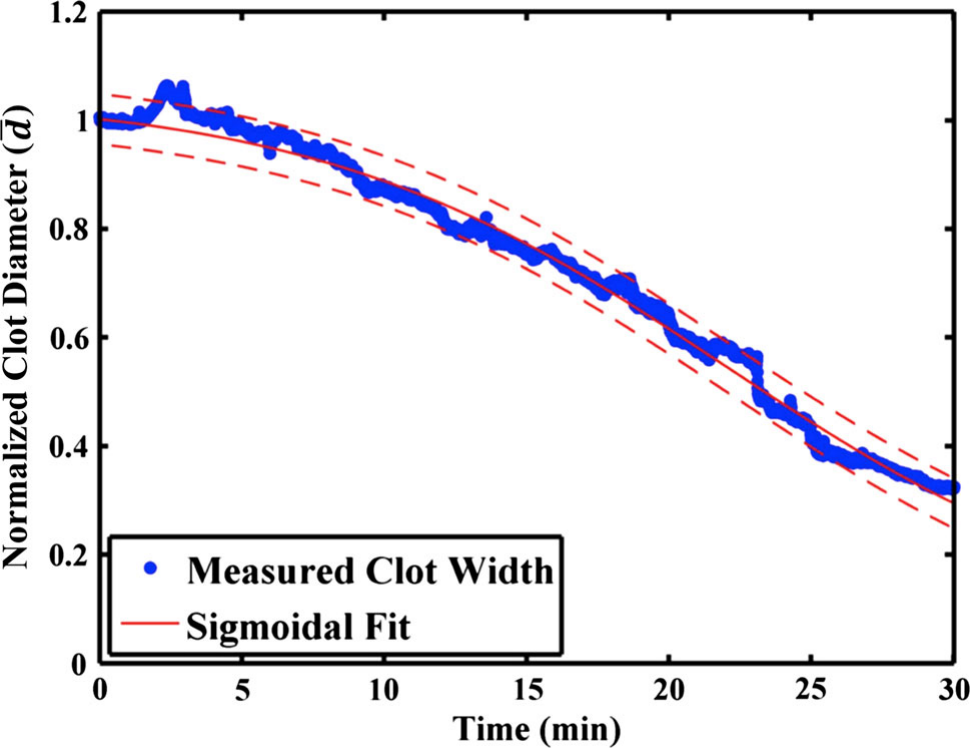
Representative reduction in clot width over the course of a 30 min treatment (120-kHz ultrasound exposure of 0.32 mg/mL rt-PA and Definity^®^). The *solid*, *red* line is the least-squares fit of the data to the sigmoidally decreasing function, Eq. (2), and the *dashed*, *red* *lines* are the 95 % confidence intervals of the fit to the data

**Fig. 3:**
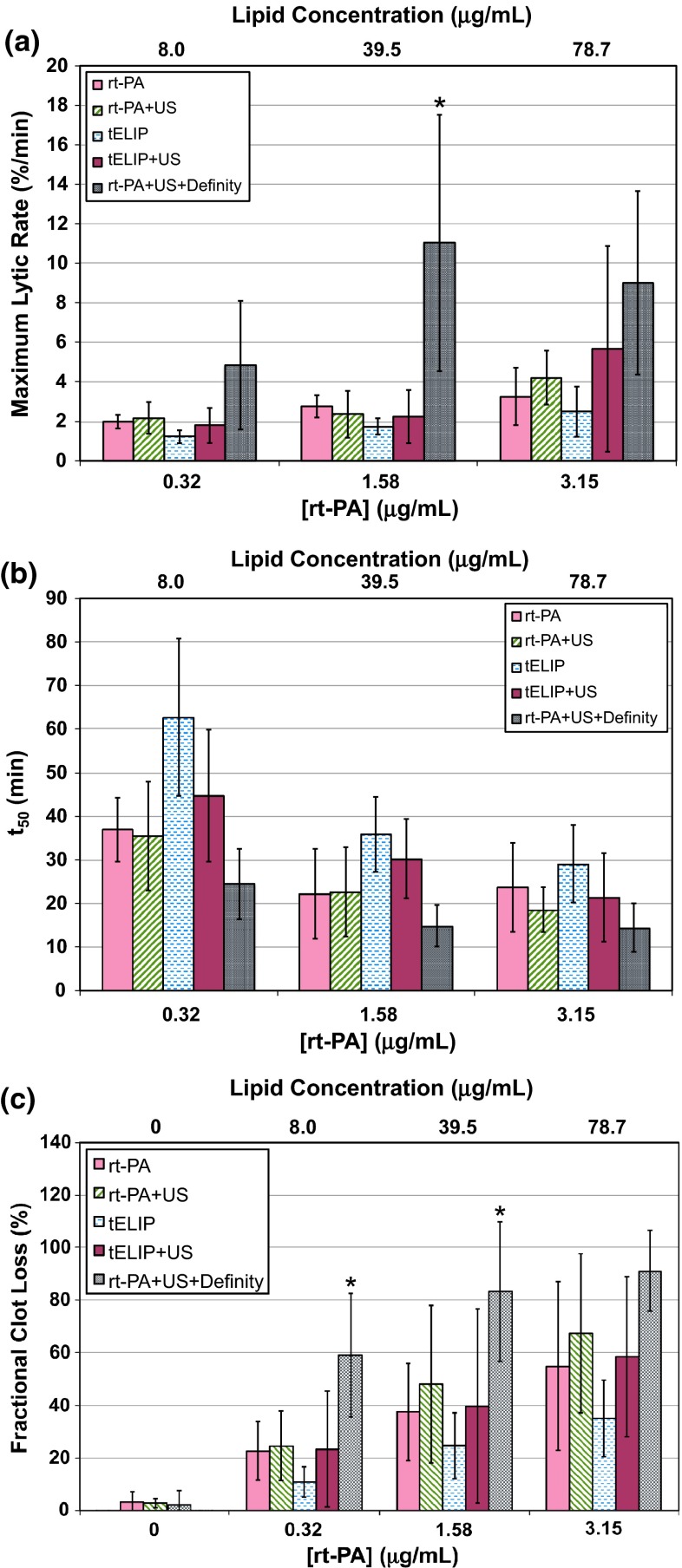
Thrombolytic efficacy data for rt-PA, rt-PA and Definity, and t-ELIP at each target concentration of rt-PA, with and without intermittent 120-kHz ultrasound exposure (US). **a** Maximum lytic rate for all treatment arms determined using Eq. (3), with *p* values indicated in Table 1. **b** Time to 50 % clot width reduction, determined from Eq. (2), with *p* values indicated in Table 2. **c** Fractional clot loss at the completion of the treatment (30 min), using Eq. (1), with *p* values indicated in Table 3. The *asterisks* (*) shown in **a** and **c** indicate the treatment arm is greater (*p* < 0.05) than all other treatment arms for that particular rt-PA concentration. The lipid concentration of t-ELIP is indicated on the top abscissa label. The sample size for each treatment (*n*) is 12

A representative example for the reduction in clot width over the 30 min treatment period, along with the least squares fit of the sigmoidal decrease given by Eq. (2), is shown in Fig. 2. The coefficient of determination (*r* ^2^) for the sigmoidal function was 0.84 ± 0.21 for all the data sets, indicating a good fit. In the absence of rt-PA, the clot width was not well described by a sigmoidal decrease (*r* ^2^ = 0.59 ± 0.30). Thus, only the fractional clot loss is reported for the thrombolytic efficacy of data acquired in the absence of rt-PA (see Figs. 3c, 6c).

### Maximum lytic rate and time to 50 % reduction in clot width

The maximum lytic rates, defined in Eq. (3), for each treatment type are shown in Fig. 3a, and corresponding *p* values between the groups are reported in Table 1. Ultrasound exposure of rt-PA, and Definity^®^ had the largest maximum lytic rate on average at all concentrations of rt-PA. However, it was only larger than all groups at 1.58 μg/mL rt-PA. The time to 50 % reduction in clot width, the fitting parameter $t_{50}$ in Eq. (2), was lowest on average for rt-PA and Definity^®^ exposed to 120-kHz ultrasound, as shown in Fig. 3b. Table 2 shows that $t_{50}$ for 120-kHz ultrasound exposure of rt-PA and Definity^®^ was not significantly lower than rt-PA alone, or 120-kHz ultrasound exposure of rt-PA. The maximum lytic rate significantly increased with rt-PA concentration for all cohorts except rt-PA and Definity^®^ exposed to 120-kHz ultrasound. The time to 50 % reduction in clot width significantly decreased with rt-PA concentration for all cohorts except rt-PA alone. The maximum lytic rate and $t_{50}$ were not reported in the absence of rt-PA due to the poor fit of Eq. (2) to the clot width reduction.

**Table 1:** The Tukey’s HSD test adjusted *p* values comparing the treatment arms for the maximum lytic rate, as shown in Fig. 3a

	rt-PA	rt-PA + US	tELIP	tELIP + US
0.32 μg/mL				
rt-PA + US	1.00	–	–	–
tELIP	0.95	0.90	–	–
tELIP + US	1.00	1.00	0.84	–
rt-PA + US + Definity	0.06	0.08	**0.00**	**0.03**
1.58 μg/mL				
rt-PA + US	1.00	–	–	–
tELIP	0.95	0.99	–	–
tELIP + US	0.99	1.00	1.00	–
rt-PA + US + Definity	**0.00**	**0.00**	**0.00**	**0.00**
3.15 μg/mL				
rt-PA + US	1.00	–	–	–
tELIP	1.00	1.00	–	–
tELIP + US	1.00	1.00	0.35	–
rt-PA + US + Definity	**0.02**	0.16	**0.00**	0.36

Bold values indicate a significant difference between the treatment arms

**Table 2:** The Tukey’s HSD test adjusted *p* values comparing the treatment arms for the time to 50 % clot width reduction, as shown in Fig. 3b

	rt-PA	rt-PA + US	tELIP	tELIP + US
0.32 μg/mL				
rt-PA + US	1.00	–	–	–
tELIP	**0.02**	**0.01**	–	–
tELIP + US	0.83	0.72	0.08	–
rt-PA + US + Definity	0.45	0.56	**0.00**	**0.02**
1.58 μg/mL				
rt-PA + US	1.00	–	–	–
tELIP	0.06	0.10	–	–
tELIP + US	0.28	0.42	0.73	–
rt-PA + US + Definity	0.56	0.57	**0.00**	**0.01**
3.15 μg/mL				
rt-PA + US	0.99	–	–	–
tELIP	0.99	0.88	–	–
tELIP + US	0.57	0.92	0.21	–
rt-PA + US + Definity	**0.02**	0.11	**0.00**	0.21

Bold values indicate a significant difference between the treatment arms

### Fractional clot loss

The fractional clot loss (*FCL*), defined by Eq. (1), for each treatment type is shown in Fig. 3c. The FCL was typically less than 5 % for all treatment arms in the absence of rt-PA. As indicated in Table 3, 120-kHz ultrasound exposure of rt-PA and Definity^®^ typically yielded a significantly larger *FCL* than all cohorts. Ultrasound exposure did not significantly increase the *FCL* of t-ELIP over t-ELIP alone. Similarly, ultrasound exposure did not significantly increase the *FCL* of rt-PA compared to rt-PA alone. The *FCL* of t-ELIP and ultrasound exposure was not significantly larger than rt-PA alone at any concentration of rt-PA. The *FCL* increased with concentration of rt-PA for all treatment types.

**Table 3:** The Tukey’s HSD test adjusted *p* values comparing the treatment arms for the fractional clot loss, as shown in Fig. 3c

	rt-PA	rt-PA + US	tELIP	tELIP + US
0 μg/mL				
rt-PA + US	0.99	–	–	–
tELIP	–	–	–	–
tELIP + US	–	–	–	–
rt-PA + US + Definity	0.86	0.93	–	–
0.32 μg/mL				
rt-PA + US	1.00	–	–	–
tELIP	0.58	0.44	–	–
tELIP + US	1.00	1.00	0.37	–
rt-PA + US + Definity	**0.00**	**0.00**	**0.00**	**0.00**
1.58 μg/mL				
rt-PA + US	0.87	–	–	–
tELIP	0.72	0.18	–	–
tELIP + US	1.00	0.92	0.46	–
rt-PA + US + Definity	**0.00**	**0.02**	**0.00**	**0.00**
3.15 μg/mL				
rt-PA + US	0.75	–	–	–
tELIP	0.25	**0.02**	–	–
tELIP + US	0.99	0.87	0.06	–
rt-PA + US + Definity	**0.01**	0.19	**0.00**	**0.01**

Bold values indicate a significant difference between the treatment arms

### Cavitation emissions

**Fig. 4:**
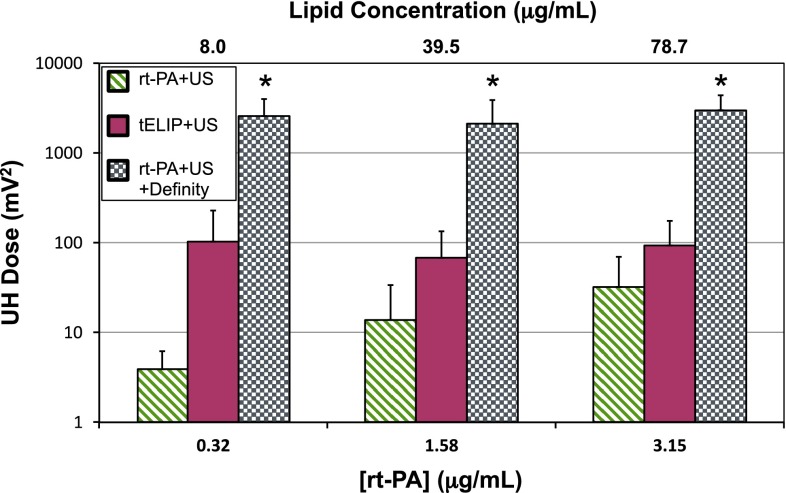
The ultraharmonic dose, defined as the summation of ultraharmonic emissions over the 30 min treatment duration, shown for all cohorts employing 120-kHz insonation at all concentrations of rt-PA. The *asterisk* (*) indicates the treatment arm is significantly greater (*p* < 0.05) than all other treatment arms for a given concentration of rt-PA. The lipid concentration of t-ELIP is indicated on the top abscissa. The sample size for each treatment (*n*) is 12

The UH dose from exposure to 120-kHz ultrasound of rt-PA, or rt-PA and Definity^®^, or t-ELIP is shown in Fig. 4. Definity^®^ produced larger UH emissions at all concentrations of rt-PA. Insonation of t-ELIP produced larger UH emissions than insonation of rt-PA, except at the highest concentration of rt-PA (3.15 μg/mL). However, the UH dose of t-ELIP was still two-orders of magnitude less than Definity^®^ at each concentration of rt-PA. The UH dose increased as a function of rt-PA concentration for insonation of rt-PA alone, but not t-ELIP or rt-PA and Definity^®^.

### rt-PA activity

**Fig. 5:**
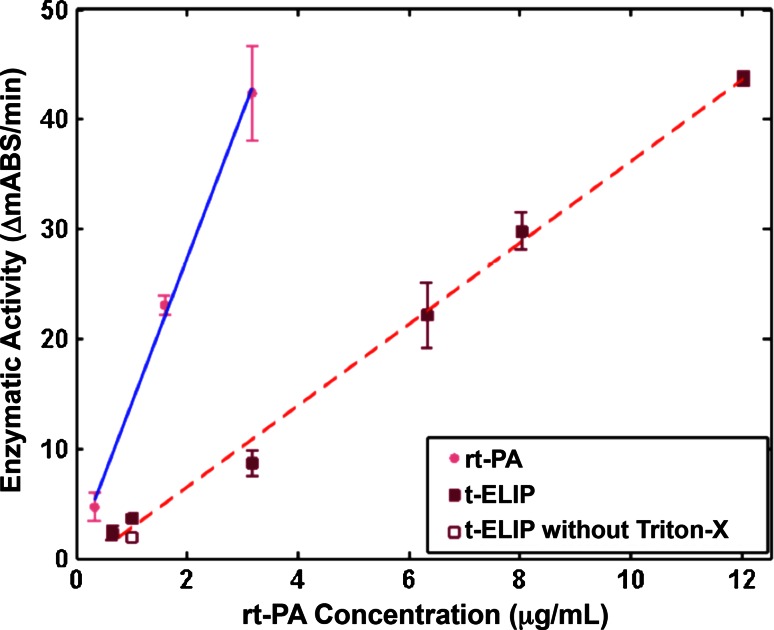
Enzymatic activity of rt-PA released from t-ELIP (*solid squares*) and rt-PA (*solid circles*). The *solid*, *blue* *line* is the linear fit of activity for rt-PA as a function of rt-PA concentration, and the *dashed*, *red* *line* is the linear fit of activity for t-ELIP as a function of rt-PA concentration. For reference, the activity of t-ELIP without the addition of Triton-X in the spectrophotometric assay is shown 1 μg/mL rt-PA (*open square*), and is approximately 59 % of that when Triton-X is used

The rt-PA enzymatic activity as a function of rt-PA concentration for rt-PA and t-ELIP is shown in Fig. 5. The encapsulation efficiency of t-ELIP, determined by comparing the enzymatic activity in the absence and presence of Triton-X at 1 μg/mL, was 59 %. The activity of both rt-PA and t-ELIP depended linearly on the target concentration of rt-PA (*r* ^2^ = 0.98 for both t-ELIP and rt-PA). However, the slope of t-ELIP was smaller than that of rt-PA (3.71 ± 0.23 ΔmAbs/min for t-ELIP vs. 13.24 ± 1.77 ΔmAbs/min for free rt-PA). Thus, the activity of rt-PA from the t-ELIP solution was approximately 28 % that of rt-PA for a given concentration of rt-PA.

### FCL comparison for equal activities

**Fig. 6:**
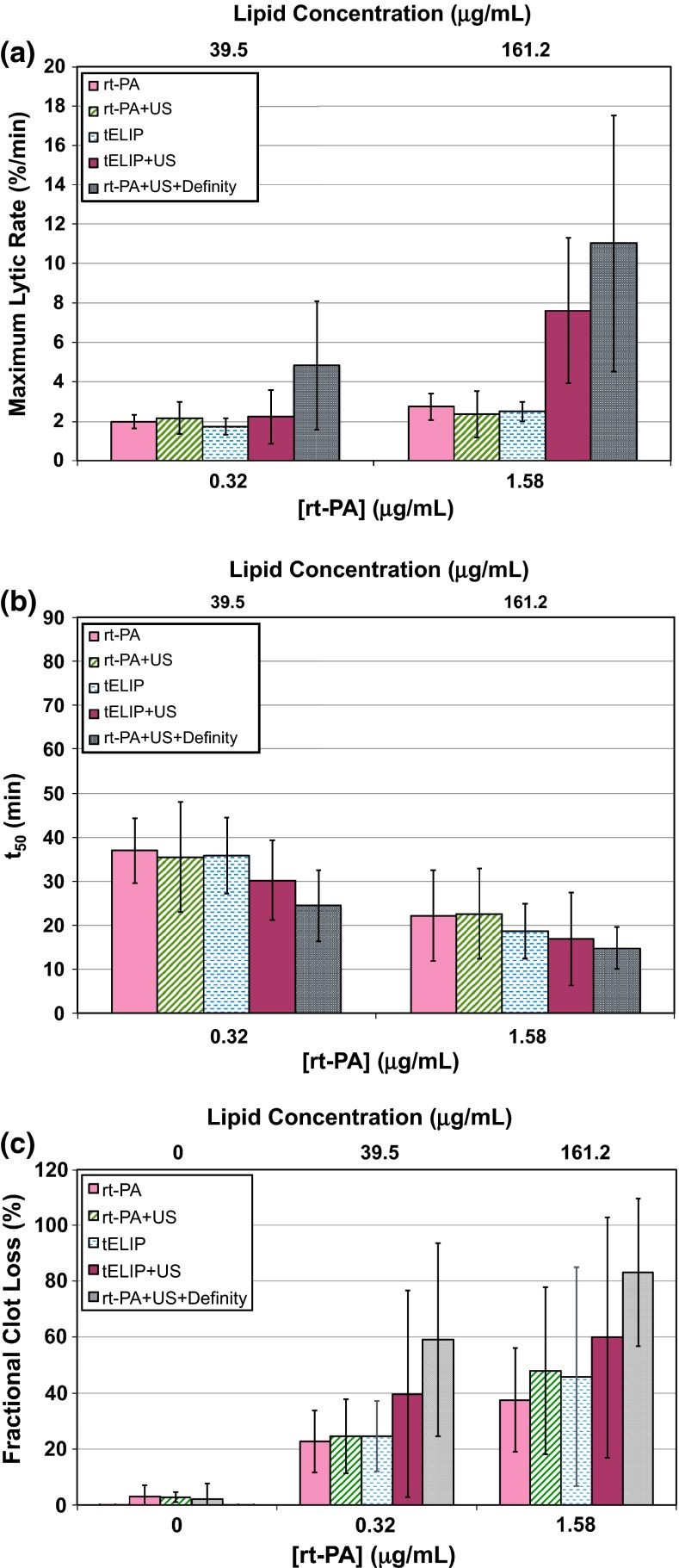
Thrombolytic efficacy data for rt-PA, rt-PA and Definity, and t-ELIP at equivalent enzymatic activity of rt-PA, with and without intermittent 120-kHz ultrasound exposure (US). **a** Maximum lytic rate for all treatment arms determined using Eq. (3 **)**, with *p* values indicated in Table 4. **b** Time to 50 % clot width reduction, determined from Eq. (2), with *p* values indicated in Table 5. **c** Fractional clot loss at the completion of the treatment (30 min), using Eq. (1), with *p* values indicated in Table 6. None of the treatments are statistically different than the all of the other treatment arms for each rt-PA concentration and thrombolytic metric shown in **a**, **b**, and **c**. The lipid concentration of t-ELIP is indicated on the top abscissa label. The sample size for each treatment (*n*) is 12

Additional data sets were taken with the t-ELIP concentration adjusted to give equivalent activity of rt-PA activity at 0.32 and 1.58 μg/mL based on the activity measurements shown in Fig. 5. The initial lytic rate, maximum lytic rate, time to 50 % reduction in clot width, and fractional clot loss at 30 min are shown in Fig. 6, and corresponding *p* values are shown in Tables 4, 5 and 6. In general, 120-kHz ultrasound exposure of rt-PA and Definity^®^ cohort exhibited maximal thrombolysis (i.e. greatest FCL, maximum lytic rate, and shortest time to 50 % reduction in clot width), but was not more efficacious than insonation of t-ELIP. However, the thrombolytic efficacy of t-ELIP exposed to ultrasound was still not higher than rt-PA alone for all thrombolytic metrics except the maximum lytic rate. The UH dose of t-ELIP with equivalent activity to rt-PA was less than Definity^®^ and greater than rt-PA at all concentrations of rt-PA.

**Table 4:** The Tukey’s HSD test adjusted *p* values comparing the treatment arms for the maximum lytic rate for equivalent enzymatic activity of rt-PA, as shown in Fig. 6a

	rt-PA	rt-PA + US	tELIP	tELIP + US
0.32 μg/mL				
rt-PA + US	0.99	–	–	–
tELIP	1.00	1.00	–	–
tELIP + US	0.98	1.00	0.98	–
rt-PA + US + Definity	**0.04**	0.11	**0.03**	**0.04**
1.58 μg/mL				
rt-PA + US	1.00	–	–	–
tELIP	1.00	1.00	–	–
tELIP + US	0.09	0.10	0.26	–
rt-PA + US + Definity	**0.00**	**0.00**	**0.02**	0.46

Bold values indicate a significant difference between the treatment arms

**Table 5:** The Tukey’s HSD test adjusted *p* values comparing the treatment arms for the time to 50 % clot width reduction for equivalent enzymatic activity of rt-PA, as shown in Fig. 6b

	rt-PA	rt-PA + US	tELIP	tELIP + US
0.32 μg/mL				
rt-PA + US	1.00	–	–	–
tELIP	1.00	1.00	–	–
tELIP + US	0.91	0.83	0.75	–
rt-PA + US + Definity	0.31	0.22	0.15	0.57
1.58 μg/mL				
rt-PA + US	1.00	–	–	–
tELIP	1.00	0.97	–	–
tELIP + US	0.92	0.80	1.00	–
rt-PA + US + Definity	0.72	0.55	0.97	0.99

**Table 6:** The Tukey’s HSD test adjusted *p* values comparing the treatment arms for fractional clot loss for equivalent enzymatic activity of rt-PA, as shown in Fig. 6c

	rt-PA	rt-PA + US	tELIP	tELIP + US
0 μg/mL				
rt-PA + US	0.99	–	–	–
tELIP	–	–	–	–
tELIP + US	–	–	–	–
rt-PA + US + Definity	0.86	0.93	–	–
0.32 μg/mL				
rt-PA + US	1.00	–	–	–
tELIP	1.00	1.00	–	–
tELIP + US	0.37	0.49	0.41	–
rt-PA + US + Definity	**0.01**	**0.02**	**0.01**	0.25
1.58 μg/mL				
rt-PA + US	0.93	–	–	–
tELIP	0.97	1.00	–	–
tELIP + US	0.43	0.90	0.83	–
rt-PA + US + Definity	**0.01**	0.07	**0.05**	0.40

Bold values indicate a significant difference between the treatment arms

## Discussion

An in vitro model and spectrophotometric assay were employed to assess stable cavitation emissions and thrombolytic efficacy associated with several ultrasound-enhanced treatment schemes. The functional form for clot width reduction was found to be better described by a sigmoidally decreasing function, Eq. (2), than an exponentially decreasing function, such as that employed by Meunier et al. [29]. Bajd et al. [30] also noted that the instantaneous reduction in clot area was sigmoidal in the presence of flow. The same group also developed a molecular dynamics model to investigate numerically blood clot dissolution [31]. Their findings provided theoretical confirmation of the sigmoidal decrease of thrombus area exposed to rt-PA under flow.

Exposure of t-ELIP to 120-kHz ultrasound had equivalent thrombolytic efficacy to rt-PA in flow, regardless of concentration. Shaw et al. [6] found a strong enhancement of the fractional clot loss for pulsed (80 % duty cycle) 120-kHz exposure of t-ELIP over rt-PA exposed to pulsed ultrasound. Similarly, Hua et al. (2014) found an increased efficacy rate for 2-MHz insonations of rt-PA loaded microbubbles compared to insonation of rt-PA and microbubbles in a rabbit thrombus model. Shaw et al. employed a stasis model and had considerably smaller fractional clot loss for the rt-PA alone treatment compared to the present flow study (30 % for Shaw et al. vs. 55 % in the present study, Fig. 3c). Larger fractional clot losses were found when t-ELIP was exposed to pulsed 120-kHz ultrasound compared to rt-PA alone in the study of Shaw et al. (45 % for Shaw et al. vs. 30 % for the present study, Fig. 3c). In the present study, t-ELIP without ultrasound exposure had a lower fractional clot loss than rt-PA alone. It is unknown if the addition of flow to the in vitro model would affect the uptake of t-ELIP to the thrombus. Regardless, flow appears to be a significant component of the thrombolytic efficacy of t-ELIP.

Laing et al. [14] found a significant increase in the maximum percent recanalization of an in vivo rabbit aortic thrombus model treated with t-ELIP, or t-ELIP and 6-MHz duplex (color) Doppler compared to PBS controls. Similarly, the fractional clot loss, shown in Figs. 3c and 6c, increased at all concentrations of t-ELIP compared to plasma controls, except at 0.32 μg/mL rt-PA (8 μg/mL lipid). Laing et al. found no difference in thrombolytic enhancement of t-ELIP alone versus t-ELIP exposed to 6-MHz Doppler ultrasound. The data presented in Figs. 3 and 6 is consistent with their findings (*p* values are provided in Tables 1, 2, 3, 4, 5, 6). Laing et al. found no difference between rt-PA and Definity^®^ or rt-PA or t-ELIP exposed to 6-MHz Doppler ultrasound. Like the Laing study, the present study also showed no difference between rt-PA or t-ELIP with or without exposure to ultrasound. However, the present study demonstrated increased thrombolytic efficacy for rt-PA and Definity^®^ exposed to 120-kHz ultrasound in vitro, which is likely due to the insonation scheme used to promote sustained bubble activity.

The UH dose of t-ELIP was less than Definity^®^, and only barely more than the UH dose obtained with rt-PA alone (Fig. 4). In addition, although an increased concentration of t-ELIP was used to match the rt-PA enzymatic activity, the ultrasound dose did not increase despite the increased number of cavitation nucleation sites [32]. UH emissions are known to correlate with the thrombolytic efficacy during sonothrombolysis [10, 27]. Liberated gas from Definity^®^ has previously been observed to coalesce during exposure to 120-kHz ultrasound, resulting in resonant-sized (50 μm), long-lived (>100 s) microbubbles [10]. Such acoustically active microbubbles were not observed during insonation of t-ELIP.

The reduced cavitation activity may be in part due to the rapid solubility of the air present in t-ELIP compared to the low solubility and low diffusivity of perfluorocarbon present in Definity^®^ [33]. The lipid shell is thought to rupture during insonation [34], leaving the gas core in direct contact with the surrounding fluid. A micrometer-sized, air-filled microbubble would dissolve into solution within a few seconds [35], which is on the order of the transient time across the clot used in these studies. In contrast, Definity^®^ microbubbles appear to be stable for tens of minutes [33]. The inclusion of a perfluorocarbon gas in t-ELIP may help to generate the sustained, stable cavitation activity necessary for thrombolytic enhancement [36]. Given the equivalent thrombolytic efficacy already achieved between t-ELIP or rt-PA and Definity^®^ exposed to 120-kHz ultrasound, further thrombolytic enhancement might be expected through incorporation of a perfluorocarbon gas in t-ELIP.

The spectrophotometric measurements of the absorbance of rt-PA are similar to those observed by Smith et al. [7] (slope of 14.8 ΔmAbs/min for Smith et al. vs. 13.34 ± 1.77 ΔmAbs/min for the present study). The 59 % entrapment efficiency of rt-PA in t-ELIP measured here is similar to that reported previously [7, 11]. The enzymatic activity of t-ELIP has previously been investigated [7, 11]. However, these studies did not directly compare t-ELIP and rt-PA at the same concentration of rt-PA. The distinct reduction of rt-PA activity from t-ELIP compared to rt-PA may be due in part to the multiple freeze/thaw steps required for manufacturing t-ELIP [37]. The enzymatic activity of proteins similar to rt-PA have been shown to be sensitive to freeze–thaw cycling [38].

**Fig. 7:**
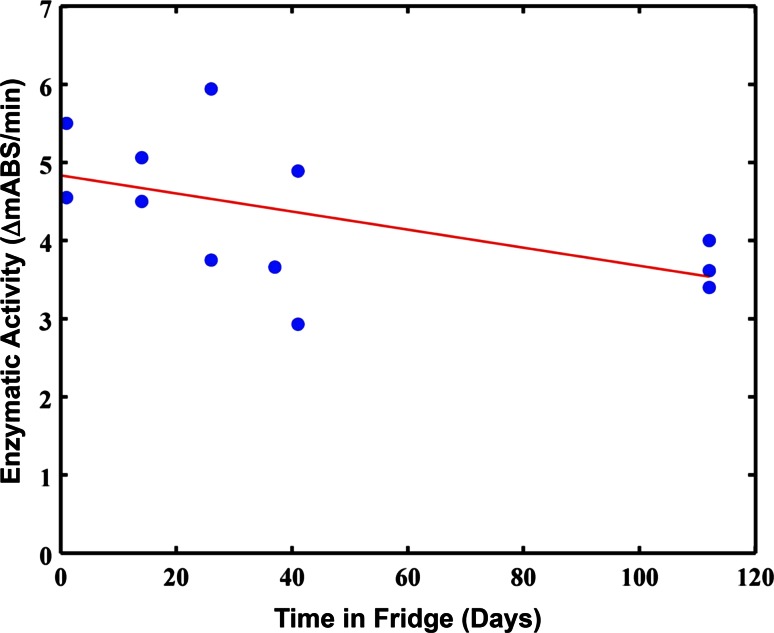
Enzymatic activity of rt-PA in t-ELIP as a function of storage at 5 °C prior to reconstitution. The *solid*, *red* line is a linear fit to the data, which has an insignificant trend towards decreasing efficacy as a function of storage date

Thrombolytic-loaded ELIP are typically stored at 5 °C until reconstitution and use, as per typical protocol for reconstitution of ELIP [32]. The activity of rt-PA is known to degrade unless stored at −20 to −80 °C [39]. Preliminary measurements of t-ELIP activity over time stored at 5 °C shows a slight but insignificant (*p* > 0.05) trend for degradation, as shown in Fig. 7. Similarly, the rt-PA used in the manufacturing of this t-ELIP was stored at 5 °C prior to incorporation into the lipid, which may have degraded the enzymatic activity [16].

The in vitro flow model used in this study allowed an in-depth study of t-ELIP thrombolytic mechanisms. However, several aspects of this in vitro study limit the applicability of these findings. The low flow rate considered in this model was fixed, and neglects the contribution of increased flow rates as the clot lyses. The additional shear stresses associated with increased flow rates, as would occur in vivo, have previously been shown to increase the lytic rate [12]. Only ambient pressure was considered in this model, which may modify the nonlinear response of microbubbles compared to hemodynamic pressures in vivo [40]. Time-varying pressure gradients are known to increase the penetration of thrombolytics in clot samples [41], thus increasing the degree of clot lysis. The clinical scenario of an occluding thrombus was not modeled here [42]. The fibrin targeting capabilities of t-ELIP to clot have previously been established in vitro [5]. Other thrombolytic schemes have targeted activated platelets [43] or endothelial-cell-surface determinants [44], and may provide specificity to thrombi instead of hemostatic plugs. However, the systemic toxicity and side effects of the treatment could also not be ascertained in this in vitro model. Further in vivo studies are needed to assess the potential reduction of adverse bioeffects in vivo.

## Summary

A complex relationship between the enzymatic activity of rt-PA and the stable cavitation activity necessary to generate enhanced thrombolysis was determined in an in vitro flow model. The use of ELIP for ultrasound-enhancement of thrombolysis requires both a potent thrombolytic and sufficient nuclei to sustain stable cavitation activity. This study demonstrated that a novel targeted rt-PA encapsulated agent, t-ELIP, is less effective than a systemic lytic with a more robust cavitation agent. To maximize the efficacy of this targeted, encapsulated agent, incorporation of an insoluble gas that promotes more sustained cavitation activity is preferred.
